# A Randomized, Crossover, Single-Dose Bioequivalence Study of Two Extended-Release Tablets of Donepezil 23 mg in Healthy Human Volunteers under Fasting and Fed States

**DOI:** 10.3797/scipharm.1302-13

**Published:** 2013-04-25

**Authors:** Chaitanya Gadiko, Sudhakar Koundinya Tippabhotla, Satyanarayana Thota, Ramakrishna Battula, Sohel Md. Khan, Venkateswarlu Vobalaboina

**Affiliations:** Clinical Pharmacology and Pharmacokinetics, Integrated Product Development, Dr. Reddy’s Laboratories Ltd., Hyderabad-500090, India.

**Keywords:** Bioequivalence, Donepezil, Pharmacokinetic, Non-compartmental

## Abstract

To assess the bioequivalence of two extended-release tablets of donepezil 23 mg, open label, randomized, single-dose, two-sequence, two-period crossover studies under fasting (n=74) and fed (n=94) conditions in healthy adult human volunteers were conducted. Subjects were randomized to either of the two treatment arms (test or reference) separated by a washout period of 28 days. Blood samples were collected up to 72 h post-dose and plasma samples were analyzed for donepezil using a validated LC-MS/MS method. Pharmacokinetic parameters were derived using a non-compartmental approach. Bioequivalence was evaluated in 69 subjects in the fasting study, and 71 subjects in the fed study. In the fasting study, the 90% CI of C_max_ and AUC_0-72_ were 82.50–90.10 and 92.38–98.60, respectively. Corresponding values in the fed study were 91.82–98.05 and 97.27–100.27. Based on the results, the test product (donepezil) met the US regulatory criteria of bioequivalence relative to the reference product (Aricept^®^) under both fasting and fed conditions.

## Introduction

Alzheimer’s Disease (AD) is a neurodegenerative disorder that primarily effects the elderly population [1]. It is characterized by progressive cognitive deterioration, together with the inability to perform daily activities and neuropsychiatric symptoms or behavioral changes [2]. In 2003, it was estimated that there were 28 million sufferers of AD worldwide and this figure is likely to increase four-fold in the next 50 years [3].

Cholinesterase inhibitors were the first line of drugs approved by the US Food and Drug Administration (FDA) for the treatment of AD [2]. They have been used either as monotherapy or in combination with other drugs like memantine (an NMDA antagonist) [4]. Donepezil is one of the most widely used drugs approved for the treatment of mild-to-moderate and severe AD. It exerts its effect by a selective and reversible inhibition of acetylcholinesterase, thereby inhibiting the hydrolysis of acetylcholine [5]. Elevated acetylcholine levels in the brain are responsible for the improvement in cognitive functioning in treated AD patients [6]. The conventional dosage forms available are immediate-release formulations (donepezil tablets and donepezil ODT) available at 5 mg and 10 mg strengths administered once a day [7]. Donepezil is dose-proportional and anticholinesterase activity is directly correlated with dose. Hence, an increase in dose is associated with an increased anticholinesterase activity which in turn may improve the cognitive performance in AD [8]. With the immediate-release dosage forms, an initial spiking of donepezil in patients’ blood was observed, which may result in unwanted side effects (CNS and GIT problems) and reduced patient compliance [9]. To overcome these undesirable side effects associated with conventional immediate-release formulations and to provide a gradual release, an extended-release (ER) formulation of donepezil 23 mg was developed and approved for treating patients of moderate-to-severe AD [10]. Following the oral administration of the donepezil 23 mg ER tablet, the time to reach the peak plasma concentration was delayed by 8 h compared to 3 to 5 h observed with the donepezil 10 mg tablets [11]. Further peak plasma concentrations were almost two-fold higher for the 23 mg tablets compared to the 10 mg tablets.

The Aricept^®^ 23 mg tablet is a branded formulation of donepezil ER tablets that is being marketed in the United States (US) [12]. A generic version of the donepezil ER tablets was developed by Dr. Reddy’s laboratory for marketing in the US. Most of the published reports on comparative bioavailability studies of donepezil were on the IR formulation (available in 5 mg and 10 mg strengths). There is no published data on either the pharmacokinetics or the comparative bioavailability in healthy subjects for the ER formulation of donepezil 23 mg. Hence, the objective of the present study was to evaluate and compare the relative bioavailability of generic and branded formulations of donepezil 23 mg ER tablets in healthy, adult human volunteers (under fasting and fed states) for the purpose of marketing the generic formulation in the US.

## Results

### Subjects

In the fasting study, of the total 76 enrolled, 70 subjects completed the clinical phase of the study and 69 subjects were included in the pharmacokinetic and statistical analysis. Five subjects (four subjects prior to dosing in period II and one subject after dosing in period I) were withdrawn because of adverse events (AEs), one subject withdrew his consent prior to dosing in period II, and one subject who completed both the periods was not included in the statistical analysis due to non-compliance with the inclusion/exclusion criteria.

In the fed study, of the total 76 enrolled, only 65 subjects completed period I of the study. Due to a higher rate of dropouts than expected, and given that period II of the study was yet to be completed, an additional group (add-on) of 18 subjects was enrolled in order to obtain sufficient information to assess the bioequivalence of donepezil. Therefore, an overall total of 94 subjects were included in the fed study and 71 subjects completed the crossover design and were included in the pharmacokinetic and statistical analysis. Out of the 23 withdrawn subjects, 19 subjects (of whom 13 subjects after dosing in period I and six subjects after dosing in period II) were withdrawn because of AEs, three subjects withdrew their consent prior to dosing in period II, and one subject met the withdrawal criteria prior to dosing in period II.

Data from the subjects who were withdrawn due to adverse events were analyzed due to safety concerns, but the data from these subjects were not considered for pharmacokinetic and statistical analysis. Demographic data of the subjects completing the bioequivalence studies are summarized in Table 1.

### Safety Results

Safety was evaluated through the assessment of AEs, standard laboratory evaluations, and vital signs. In the fasting study, a total of 154 AEs were reported by 52 subjects. Out of these, 40 subjects reported 73 AEs after the test product administration, and 43 subjects reported 81 AEs after the administration of the reference product. The severity of AEs ranged from mild to severe. Three severe AEs (back pain, breast inflammation, and headache) were observed during the study. Of all AEs, two (increased neutrophil count and oral herpes) were unexpected and possibly related to the administration of the test product. No AEs required the use of concomitant medications during the study. However, one subject took medication once the study was completed for an AE experienced during the protocol period of observation. One subject was withdrawn from the study for safety reasons.

In the fed study, 81 subjects experienced a total of 305 AEs. Of these, 53 subjects reported 115 AEs after the single-dose administration of the test product and 68 subjects (74.7%) reported 190 AEs (12 different SOCs and 47 different PTs) after the single-dose administration of the reference product. The severity of AEs ranged from mild to severe. Twelve severe AEs (test: asthenia, vomiting (three occurrences); reference: aspartate aminotransferase increased, muscle spasms, nausea, neck pain, paraesthesia, and vomiting (three occurrences)) were observed during the study. Of all AEs, four (mydriasis, oral herpes, photophobia, and pupils unequal) were unexpected and possibly related to the administration of the (test or reference) products. Four subjects experienced an AE that required the use of concomitant medications during the course of this study: one subject applied a concomitant medication for prevention prior to dosing for period I and reported it at admission of period II, and one subject (1.1%) applied a concomitant medication for prevention prior to dosing for period II and reported it at admission of period II. No subject was withdrawn from the study for safety reasons.

There were no deaths or serious adverse events reported. All of the observed AEs were considered to be resolved. Given this information, the study drugs appeared to be well-tolerated and there were no safety concerns observed.

### Pharmacokinetics and Bioequivalence

**Fig. 1:**
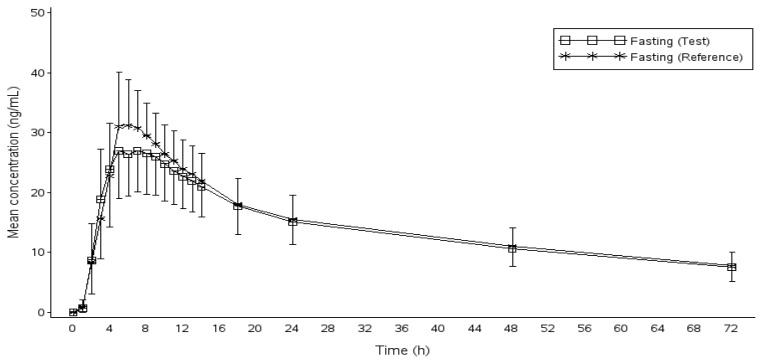
Plasma concentration-time profile of donepezil under fasting conditions (n=69).

**Fig. 2:**
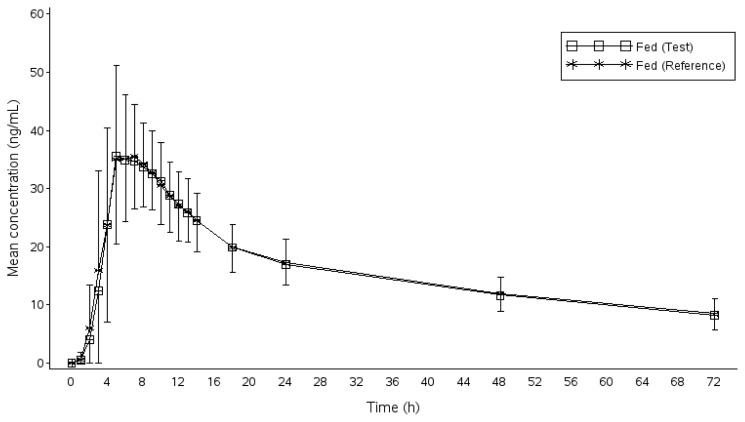
Plasma concentration-time profile of donepezil under fed conditions (n=71).

Subjects who completed both periods were included in the pharmacokinetic and statistical analysis. The actual time of sample collection was used for the calculation. The pharmacokinetic parameters of donepezil for the test and reference formulations under the fasting and fed states are tabulated in Tables 2 and 3. The mean (SD) plasma concentrations of donepezil versus time profiles for the test and reference formulations are presented in Fig. 1 (fasting study) and Fig. 2 (fed study). The curves are superimposed on each other.

The geometric least square mean (LSM) ratios and 90% confidence interval (CI) for the primary pharmacokinetic parameters [C_max_ and AUC_0-72_] of donepezil under both fasting and fed states are presented in Table 4.

The results demonstrate that the ratio and corresponding 90% CI of the relative C_max_ and AUC_0-72_ geometric LSM of the test and reference formulations were within the regulatory bioequivalence criterion of 80.00% to 125.00% under both fasting and fed conditions.

The C_max_ and AUC_0-72_ of the reference product is slightly higher than the test product with relatively no change in the median T_max_. Three subjects had positive pre-dose donepezil concentrations after the repeat analysis in period II, but these pre-dose values were less than 5% of the C_max_ value reported for each subject in period II. Hence, the subjects’ data were included in all of the pharmacokinetic measurements and calculations without any adjustments.

The intra-subject variability (ISCV) reflects the residual variability observed in the pharmacokinetic parameters after accounting for the possible differences between the sequence, period, and formulation effects as well as accounting for between-subject variations. The (%) ISCV was slightly higher under fasting conditions (15.6 and 11.4 for C_max_ and AUC_0-72_) than that observed under fed conditions (11.5 for C_max_ and 5.3 for AUC_0-72_).

ANOVA applied on the log-transformed values for C_max_ and AUC_0-72_ parameters for donepezil for the difference between all of the effects, sequences, subjects within sequences, and periods was found to be statistically insignificant (*p>*0.05).

## Discussion

The donepezil 23 mg ER formulation was developed with the aim to provide better cognitive performance when compared to the conventional immediate release 5 mg and 10 mg oral dosage forms. This was the first study to report on the comparable bioavailability of the ER formulations of donepezil 23 mg.

The objective of these studies was to compare the bioavailability and assess the pharmacokinetic profile of Dr. Reddy’s test formulation in comparison with the reference formulation. The use of a generic preparation of a therapeutically well-established active drug principle has to be justified by the appropriate bioequivalence study, because the proof of bioequivalence of the test and reference products assures equal therapeutic efficacy. To establish this, single-dose fasting and fed studies in non-smoking, healthy, adult, human subjects were planned based on the US regulatory requirements. The subject population was selected with the aim to minimize variability, and to permit detection differences between the pharmaceutical products. The choice of the drug’s strength used was justified based on analytical, pharmacokinetic, and safety grounds. Furthermore, this strength was the Reference Listed Drug (RLD) recommended by the FDA.

As bioavailability/bioequivalence studies do not typically require a double-blind study, this study design was open-label. A crossover design was planned as per the USFDA requirements. For comparative bioavailability studies, this is considered as the preferred design in order to minimize subject-by-subject variations and to minimize variability between drug treatments [13].

Multiple blood samples were collected prior to and up to 72 h after drug administration according to the study protocol. This sampling was planned in order to provide a reliable estimate of the extent of absorption [14]. Samples were processed and stored under conditions that have been shown not to cause significant degradation of the analyte. For this bioequivalence study with a crossover design, each subject (except the withdrawn subjects) received both treatments during the two periods. Hence, every subject acted as his or her own control and no separate group of subjects was required to act as the control group.

According to US FDA guideline, for a modified release formulation, if subjects experience emesis at any time during the labeled dosing interval (i.e., 24 h), the data from the subject should be excluded from the statistical analysis [15]. Donepezil has been shown to produce severe nausea and vomiting and the mechanism is thought to be through central- and peripheral-mediated action of acetylcholine on muscarinic receptors. Because of this side effect (emesis), the subjects need to be withdrawn from the study in case he/she vomits at any time during the study period. To overcome this, there were many approaches adopted by physicians in the study centers. Researchers have used various antiemetic drugs to counteract the nausea and vomiting observed with anticholinesterases [16–18]. The choice of an appropriate antiemetic depends upon the pharmacology of the study medication, study objectives, chemistry, physical properties etc.

F Fu *et al.* evaluated the ability of the antiemetic propantheline bromide co-administered with donepezil in cerebral ischemic mice for alleviating the peripheral cholinergic side effects of donepezil without affecting its central anti-amnestic property. Propantheline bromide, an antimuscarinic drug, decreased the increase in gastric emptying and gastrointestinal motility resulting from donepezil in cerebral ischemic mice with no change in brain acetylcholinesterase (AChE) activity or latency period for the mice to escape. Propantheline bromide, being a quaternary amine, is completely ionized at physiological pH because only negligible amounts cross the blood-brain barrier and enter the CNS. From this preclinical experimentation in cerebral ischemic mice, he suggested that propantheline bromide is unable to enter the CNS in significant amounts and can alleviate the peripheral cholinergic side effects of anticholinesterases such as donepezil without affecting its central anticholinergic activity [19]. We evaluated the ability of propantheline bromide in ameliorating the vomiting in study subjects in our pilot bioequivalence trial. Unfortunately, the abolition rate was low and subjects did experience vomiting even after prior administration of propantheline bromide. Therefore, a transdermal scopolamine patch can be applied prior to donepezil administration to provide a blockade of cholinergic adverse events related to donepezil.

Scopolamine is a naturally occurring belladonna alkaloid that has been found to be highly effective in the prevention of symptoms from motion sickness such as nausea and vomiting. It is a centrally-active anticholinergic drug that exerts its effect through competitive antagonism of acetylcholine (or other direct cholinomimetics) at the muscarinic receptor, and thus blocks the vestibular input to the emetic center. This means that a high dose of a cholinomimetic agent is required to abolish its effect [20].

The effectiveness of scopolamine in motion sickness is thought to act by blocking cholinergic activity from the vestibular center to the central nervous system (CNS), thereby blocking the reflex action of vomiting. In addition, a direct action on the vomiting centre may also be involved. After transdermal application of a scopolamine disc, peak levels were reached at approximately 12 h and the drug release was continuous until a time of 72 h [21].

The pharmacokinetic parameters evaluated for donepezil under fasting and fed states were in accordance with the literature values. Even though there was no significant effect from food on the bioavailability of donepezil, the study was conducted under both fasting and fed conditions to meet the US regulatory requirement. In line with the literature, there was a slight increase in the C_max_ and AUC_0-72_ following the single-dose administration of donepezil 23 mg tablets in the fed state compared to the fasting state. Also, the incidence of vomiting was greater in the fed study compared to the fasting study [22].

In the fasting study, taking into account the concentration values reported for the last blood draw in period I and the 28-day washout period, it is unlikely that residual drug carryover effects influenced the results of the second period for the three subjects who had pre-dose donepezil concentrations in period II. Furthermore, these subjects did not report any intake of donepezil during the washout period. Finally, these pre-dose values were less than 5% of the C_max_ value reported for each subject in period II, and should not influence bioequivalence assessment. Hence, the data of these subjects were included in the pharmacokinetic analysis without any adjustments.

ANOVA applied on the log-transformed values for the C_max_ and AUC_0-72_ parameters for donepezil under the fasting and fed states for the difference between all the effects, namely treatment, sequence, and period, were found to be statistically insignificant (p>0.05), indicative of the absence of significant differences between the test and reference formulations.

## Conclusions

The test/reference ratio of the geometric LSMs and corresponding 90% confidence interval for the C_max_ and AUC_0-72_ were all within the acceptance range of 80.00 to 125.00%. Therefore, the test formulation (donepezil hydrochloride 23 mg extended release tablets, Dr. Reddy’s Laboratories Limited, India) was judged to be the bioequivalent to the reference formulation (Aricept^®^ 23 mg tablets, Eisai Inc., USA) under both fasting and fed conditions. Overall, the drug was tolerated because of the adjunction of a concomitant medication; alone this drug at this dosage is poorly tolerated.

## Experimental

### Study Design

Two separate studies were performed and the studies were designed as an open-label, randomized, two-period, two-treatment, two-sequence, two-way crossover, bioequivalence study in healthy adult volunteers under fasting and fed conditions.

### Study Subjects and Ethics

Male and female volunteers, light, non- or ex-smokers, aged between 18 to 45 years old and body mass index between 21 to 30 kg/m^2^ were enrolled. The screening procedure was conducted within 28 days prior to the study enrollment (day one of the study). All of the subjects had given their written informed consent prior to study initiation. The studies were carried out in accordance with the in-house standard operating procedures, Tri-Council Policy Statement (Canada), ICH (International Conference on Harmonization) E6 ‘Guideline for Good Clinical Practice,’ and the principles enunciated in the Declaration of Helsinki (revised version of Seoul, Korea, 2008) [23]. The studies also met the requirements of the U.S. Code of Federal Regulations (Title 21, Part 56), the Directive 2001/20/EC (Europe), and the Tri-Council Policy Statement (Canada). The studies were conducted after prior approval of the study protocols and study-related documents by the Institutional Review Board (IRB).

Subjects were in good health as determined by a medical history, physical examination (including vital signs), electrocardiogram (12-lead ECG), and the usual clinical laboratory tests (hematology, biochemistry, urinalysis) including negative HIV, Hepatitis B, and Hepatitis C tests as well as a negative screening for alcohol and drug abuse in the urine and a negative pregnancy test (for female subjects). Subjects were instructed to abstain from consuming any alcoholic products, xanthine-containing foods and/or beverages (like chocolate, tea, coffee, cola drinks), grapefruit juice, smoking, and chewing tobacco containing-products from 24 h prior to dosing, till the last sample collection in each study period. They were also instructed to avoid using any medicine for at least 30 days prior to the first study drug administration and until study completion.

### Drug administration and study restrictions

In both fasting and fed studies, a 1.5 mg transdermal dose of scopolamine (one adhesive disc) was placed behind the ear of each volunteer about 9 h prior to donepezil administration to provide blockade of adverse events related to the cholinergic effect of donepezil. The disc was removed approximately 24 h after the donepezil administration in each period.

In fasting study, after an overnight fast of at least 10 h, subjects were administered a single oral dose of either test or reference product with 240 mL of water in sitting posture as per randomization schedule by the trained study personnel in each period. In the fed study, the test and reference products were administered in similar way after consumption of high fat high calorie breakfast (518 calories of fat, 284 cal of carbohydrates and 136 cal of protein) [25] which was served 30 min prior to dosing in each period. The composition of high fat high calorie breakfast is the same in both the periods. Compliance to the treatment administration was assessed by conducting a thorough examination of the oral cavity by trained personnel. A washout period of 28 days was allowed between the two treatments. Subjects were instructed to remain in bed in a semi reclined position for at least the first 6 h after dosing in each period. They were instructed to avoid any strenuous activity throughout their housing period. Drinking water was not allowed from 1 h pre dose to 1 h post dose, except for the 240 mL of water given during product administration. Prior to and thereafter, water will be allowed at all times. Standard meals were served at appropriate interval during the study periods.

### Blood sample collection

A total of 19 blood samples each of 4 mL (including pre-dose sample in duplicate) were collected from each subject in each period. The venous blood samples were withdrawn at pre-dose (0.0) and at 1, 2, 3, 4, 5, 6, 7, 8, 9, 10, 11, 12, 13, 14, 18, 24, 48 and 72 h following drug administration into vacutainers containing K_2_EDTA as anticoagulant. The samples were centrifuged at 1500g and 4°C for 10 min to separate plasma and then the separated plasma samples were stored at −20°C until analysis.

### Analytical method

The plasma concentrations of donepezil were assayed using a validated LC-MS/MS method with donepezil-D7 as internal standard. Bioanalytical method was validated for specificity (which includes specificity, specificity in presence of concomitantly administered compounds (ex. scopolamine), carryover), sensitivity, linearity, precision and accuracy, matrix effect, dilution integrity, recovery and stability. As scopolamine is co-administered with donepezil, the method quantifying donepezil has been verified for potential interference from scopolamine and its metabolites described in the literature [26]. Since scopolamine and its metabolites do not have the same molecular weight as donepezil and the method uses MS/MS detection, there is no interference from these compounds. However by possible in-source/interface loss of water, few metabolites may isotopically contribute to donepezil molecular weight at a definite %. However, based on scopolamine fragmentation pathway found in literature, it is observed that scopolamine metabolites do not produce same mass transition as the mass transitions used for donepezil detection. Therefore, it was concluded that scopolamine and its metabolites do not have an impact on donepezil quantification. Furthermore, as a co-administered drug, scopolamine was evaluated as part of the method development and was found to have no significant impact on donepezil quantification.

During analysis, quality control samples were distributed throughout each batch of study samples analyzed. The analysts performing the assay were kept blinded of the sequence of administration of the test (T) and reference (R) products during the entire study period. Liquid liquid extraction method was used. Calibration curve was established by using a 9-point calibration curve and 4 quality control concentrations (high (HQC), medium (MQC), low (LQC) and LOQ QC) were used during assay of samples. The linearity of the assay during method validation and project sample analysis was 0.250 to 50.000 ng/mL with limit of quantitation (LOQ) of 0.250 ng/mL.

The precision (%) for the calibration curve standards ranged from 0.8 to 2.0 (during method validation) and 3.3 to 1.9 (during project sample analysis). Corresponding accuracy values (%) range from 101.4 to 104.0 (during method validation) and 102.0 to 99.7 (during project sample analysis).

The precision (%) for all the quality control samples (HQC, MQC, LQC and LOQ QC) during method validation were 3.1, 2.4, 5.8 and 9.2 respectively. Accuracy values were 99.7, 100.0, 104.2 and 104.7 respectively. Corresponding precision (%) for all the QC samples during project sample analysis were 2.4, 3.0, 2.6 and 7.6 respectively. Accuracy values (%) were 100.5, 101.9, 102.5and 100.5 respectively. Accuracy and precision values were within the regulatory limits. This ensures that the subject sample data reported is reliable.

The Incurred Sample Reproducibility (ISR) evaluation of donepezil in human plasma was successfully assessed using HPLC with MS/MS detection. At least 10% of the individual subject samples were re-assayed and compared to the original values. At least two-third of the total samples selected for ISR evaluation have met the percent difference criteria between original and re-assayed concentrations.

### Safety and tolerability evaluation

Safety was assessed from the screening period to the end of the study. It was assessed through medical history, physical examination, vital signs assessment, 12-lead Electrocardiogram (ECG), chest X-ray, clinical laboratory parameters (e.g. serum chemistry, hematology, serology and urine analysis) at the time of screening, monitoring of adverse events, vitals measurement and subjective symptomology during the study.

### Pharmacokinetic and statistical analysis

The pharmacokinetic parameters were calculated individually for each analyzed subject from the plasma-time profile by non-compartmental approach with a log-linear terminal phase assumption using WinNonlin^®^ version 5.3 (Pharsight Corporation, USA) for donepezil. The pharmacokinetic parameters included maximum measured plasma concentration (C_max_), time to reach maximum concentration (T_max_) and area under the plasma concentration time curve from time zero to the last quantifiable concentration (AUC_0-72_).

The maximum measured plasma concentration C_max_, for the plasma concentration time profile was determined observationally as the peak concentration for each subject in each treatment. The time of maximum concentration, T_max_, was determined as the time corresponding to C_max_. AUC_0-72_ was the area under the plasma concentration versus time curve, from time zero to the last measurable time point (72 h) calculated using the linear trapezoidal method.

The intra-subject variability reflects the residual variability observed in the pharmacokinetic parameters after accounting for possible differences between sequence, period and formulation effects as well as accounting for between-subjects variations. Intra-subject coefficient of variation was calculated using the formula $\sqrt{{\text{e}}^{\text{MSE}}-1}$, where MSE is the mean square error obtained from the ANOVA model of the ln-transformed parameters. Statistical inferences were based on log-transformed values for C_max_ and AUC_0-72_ parameters. Bioequivalence of the test product with that of the reference product under fasting and fed conditions was concluded if the 90% confidence interval were within the acceptance range of 80.00–125.00% for log-transformed pharmacokinetic parameters C_max_ and AUC_0-72_ for donepezil.

Based on pilot study data, a sample size of seventy six subjects in both fasting and fed study was estimated to be sufficient to meet the 80.00 to 125.00% BE range at 5% level of significance with a statistical power of at least 80%. This also takes into account the variations around the estimated intra-subject CV as well as takes into account the possibility of observing drop-outs due to the predictable consequence of donepezil pharmacological properties. However, in fed study, due to a higher rate of drop-outs than expected, an additional group of eighteen subjects were enrolled in order to obtain sufficient information to assess the bioequivalence of donepezil. An overall of seventy six subjects in fasting and ninety four subjects in fed study were enrolled.

Statistical comparison of the pharmacokinetic parameters of the two formulations was carried out using SAS^®^ version 9.2 (SAS Institute, USA). Comparison of the pharmacokinetic parameters C_max_ and AUC_0-72_ of donepezil for log transformed data, with respect to the test and reference was done using a random ANOVA, with the General Linear Model (GLM) procedure. A GLM with sequence, treatment, and period as fixed effects and subject within sequence as random effects were used as basis for the analysis. Fed study is conducted in multiple groups in which the drug administration for the groups occurred on different dates, the statistical model was modified to incorporate the group effect. T_max_ was assessed using non parametric test on untransformed data. Test of fixed period, sequence and treatment effects were based on the Wilcoxon’s rank sum test (Mann-Whitney U-test).

## Figures and Tables

**Tab. 1:** Demographics of subjects included in pharmacokinetic analysis.

Demographic variable	Fasting study (n=69)	Fed study (n=71)
Age (years)		
Mean ± SD	33 ± 8	33 ± 8
Range	20–45	19–45
Sex		
Male	48 (69.6%)	54 (76.1%)
Female	21 (30.4%)	17 (23.9%)
Race		
White	59 (85.5%)	65 (91.5%)
Black	7 (10.1%)	4 (5.6%)
Others	3 (4.3%)	2 (2.8%)
BMI (Kg/m^2^)		
Mean ± SD	25.3 ± 2.2	25.7 ± 2.1
Range	21.4–29.6	21.4–29.9

SD…Standard deviation; BMI…Body mass index.

**Tab. 2:** Pharmacokinetic parameters of donepezil under fasting conditions.

Test Parameter	C_max_ (ng/mL)	T_max_ (h)	AUC_0-72_ (ng.h/mL)
Mean	29.738	6.320	991.428
SD	7.570	2.150	235.398
Min.	17.400	3.000	474.708
Max.	47.991	13.000	1652.512
Median	28.348	6.000	994.913
CV (%)	25.500	34.000	23.700

**Reference Parameter**	**C_max_(ng/mL)**	**T_max_(h)**	**AUC_0-72_(ng.h/mL)**

Mean	34.192	6.120	1033.834
SD	8.225	1.400	232.651
Min.	20.376	3.000	588.214
Max.	55.841	9.050	1586.299
Median	33.160	6.000	1011.914
CV (%)	24.100	22.900	22.500

**Tab. 3:** Pharmacokinetic parameters of donepezil under fed conditions.

Test Parameter	C_max_ (ng/mL)	T_max_ (h)	AUC_0-72_ (ng.h/mL)
Mean	43.095	5.94	1119.835
SD	8.959	1.96	220.13
Min.	20.565	2.000	736.287
Max.	62.473	11.000	1708.981
Median	41.715	5.000	1112.282
CV (%)	20.800	33.000	19.700

**Reference Parameter**	**C_max_(ng/mL)**	**T_max_(h)**	**AUC_0-72_(ng.h/mL)**

Mean	45.215	5.750	1135.244
SD	8.416	1.910	216.651
Min.	19.370	3.000	743.742
Max.	62.885	12.000	1746.010
Median	44.787	5.000	1114.215
CV (%)	18.600	33.200	19.100

**Tab. 4:** Summary statistics of donepezil

Parameter	Geometric LSM		T/R (%)	90% CI	% ISCV	% Power
	Test	Reference				
Fasting study						

C_max_ (ng/mL)	28.782	33.383	86.22	82.50–90.10	15.6	100.0
AUC_0-72_ (ng·h/mL)	963.39	1009.459	95.44	92.38–98.60	11.4	100.0

Fed study						

C_max_ (ng/mL)	42.087	44.365	94.88	91.82–98.05	11.5	100.0
AUC_0-72_ (ng·h/mL)	1096.88	1110.623	98.76	97.27–100.27	5.3	100.0
